# Non-alpine primary thyroid angiosarcoma

**DOI:** 10.20945/2359-3997000000460

**Published:** 2022-05-25

**Authors:** Nádia Mourinho Bala, Pedro Simões, José Maria Aragüés, Ricardo Veiga, Sílvia Guerra, Cristina Valadas

**Affiliations:** 1 Hospital Beatriz Ângelo Departamento de Endocrinologia Loures Portugal Departamento de Endocrinologia, Hospital Beatriz Ângelo, Loures, Portugal; 2 Hospital Beatriz Ângelo Departamento de Oncologia Loures Portugal Departamento de Oncologia, Hospital Beatriz Ângelo, Loures, Portugal; 3 Hospital Beatriz Ângelo Departamento de Patologia Loures Portugal Departamento de Patologia, Hospital Beatriz Ângelo, Loures, Portugal

## Abstract

Thyroid angiosarcoma is an extremely rare malignancy, which occurs more frequently in the alpine region, likely associated with iodine deficiency and endemic goiter. This is an aggressive neoplasm that usually harbors a poor prognosis. We report the case of a 49-year-old Portuguese female patient presenting with a large nodule in the anterior neck region, with rapid growth and associated dysphonia. The neck ultrasound showed a hypoechogenic and heterogeneous thyroid nodule, with a larger axis of 44 mm. The fine needle aspiration cytology was not conclusive, and a biopsy of the lesion was performed. The result was suggestive of a mesenchymal tumor constituted by spindle cells and vascular clefts, showing positivity for endothelial markers and negativity for thyroglobulin, calcitonin and TTF1. The chest CT scan performed before surgery showed multiple pulmonary nodules suggestive of secondary lesions. The patient was submitted to total thyroidectomy and lymph node dissection in order to relieve compressive symptoms. A diagnosis of thyroid angiosarcoma was made after histologic examination of the surgical specimen. Despite undergoing multiple lines of palliative chemotherapy, the pulmonary lesions increased in size and number. The patient died due to respiratory failure 29 months after the diagnosis. Thyroid angiosarcoma is a rare malignancy, generally with poor prognosis. In our case, the patient presented with pulmonary metastases at diagnosis, which is a negative prognostic factor. Due to its rarity, data regarding management and treatment of this disease are scarce.

## INTRODUCTION

Thyroid angiosarcoma is an extremely rare and highly aggressive malignant tumor. This entity constitutes less than 1% of all sarcomas, with a higher prevalence in alpine regions (1-3). This geographic variation might be associated with iodine deficiency and a long history of endemic goiter (1,3). Other factors, such as exposure to radiation and to vinyl chloride, have also been described as predisposing factors (3). These tumors are more frequent in females (ratio 9:3) and occur mainly between 50 and 80 years of age (4). The clinical manifestations of thyroid angiosarcoma are nonspecific and depend on the location, size, extension and existence of metastases (3). The prognosis is unfavorable, with a 5-year survival rate of 33.3%, because it tends to spread rapidly to the cervical lymph nodes, lungs and bone marrow (1,5). Distant metastases to soft tissues, brain and skin have also been described (2,6,7). To the best of our knowledge, only two other cases in Portugal have been reported in the literature thus far.

## CASE REPORT

**Figure 1:**
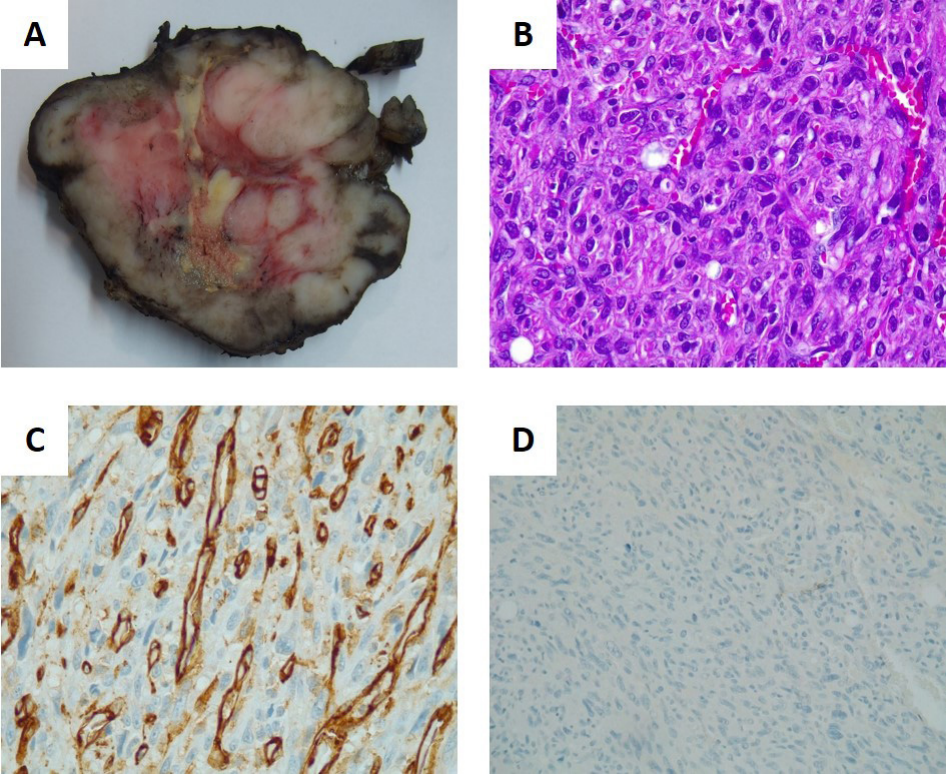
Histopathological images. (**A**) Gross examination revealing a 75 mm tumor, occupying most of the left thyroid lobe, with infiltrative, ill-defined borders and foci of necrosis. (**B**) Hematoxylin-eosin staining showing a highly vascular tumor composed of pleomorphic large cells. (**C**) Immunoreactivity for CD31 highlights the prominent tumor vasculature and shows positivity in some of the neoplastic cells. D – CK AE1/AE3 was negative throughout the tumor.

We describe the case of a 49-year-old female patient who was a smoker (40 packs/year) with no other relevant personal or family history. The patient was born in Portugal, never left the country and was referred to our department due to a 2-month history of a painless, rapidly growing mass on her anterior neck, as well as associated hoarseness. In the initial consultation, the patient presented with a palpable 4 cm nodule with hard consistency, located in the left thyroid lobe, which moved when swallowing. A cervical ultrasound was performed, showing an hypoechogenic nodule in the left thyroid lobe, with bosselated limits, measuring 44 x 43 mm. Thyroid function was normal and anti-thyroglobulin, and anti-thyroperoxidase antibodies were negative. A fine needle aspiration cytology (FNAC) was performed, revealing inflammatory cells, apoptotic nuclei and abundant fusiform cells, seemingly fibroblasts, with polymorphic nuclei and small nucleolus. These findings were regarded as inconclusive, so we obtained a needle biopsy of the lesion. The result was suggestive of a mesenchymal tumor, with fusiform cells and vascular slits, showing positivity in the immunohistochemical examination for CD31, CD34 and factor VIII. The sample was negative for thyroglobulin, calcitonin, thyroid transcription factor 1 (TTF-1) and cytokeratin AE1/AE3. Due to a high index of suspicion of malignancy, we opted to perform a cervical and thoracic computerized tomography (CT) scan in order to determine the best therapeutic approach. The CT scan showed a voluminous thyroid nodule in the left lobe, which was 52 x 50 x 66 mm, heterogeneous and had no calcifications. This lesion was causing lateral displacement of the left internal jugular vein and trachea but with no alterations in the tracheal lumen diameter. There were multiple disperse nodular lesions in the pulmonary parenchyma, suggestive of secondary lesions, the largest with a diameter of 22 mm. Due to the rapid growth of the tumor and the associated compressive symptoms, the patient was submitted to total thyroidectomy and left lymph nodes’ dissection of compartments IIA, III and IV. Gross examination revealed a 75 mm tumor, occupying most of the left thyroid lobe, with infiltrative, ill-defined borders and foci of necrosis (Figure 1). Microscopically, the tumor was highly vascular and composed of highly atypical and pleomorphic spindle cells. Necrotic foci, capsule invasion, angioinvasion and a high mitotic activity were also observed. No lymph nodes’ metastases were documented in the 29 excised lymph nodes. Immunohistochemical stains showed focal positivity for CD31 and CD34, serving as evidence of the endothelial differentiation of this tumor. On the other hand, cytokeratins, desmin, smooth-muscle actin, calcitonin and TTF-1 were all negative. The definite diagnosis of thyroid angiosarcoma (TAS) was rendered. The patient was referred to our oncology department after surgery and started palliative chemotherapy with weekly paclitaxel (80 mg/m^2^). At the second cycle (about 2 months after surgery), a nodular cervical lesion was detected, with a larger axis of 37 mm and involving the jugular vein, compatible with a local recurrence that was considered non-resectable. The patient was submitted to local radiation therapy (30 Gy, in 5 fractions) with a dimensional reduction of the mass (from a larger axis of 37 mm to 22 mm). The CT scan performed after nine cycles of paclitaxel was compatible with partial response, showing a considerable dimensional reduction in most pulmonary metastases. Despite the initial favorable response, the CT scan performed after 25 cycles of weekly paclitaxel (about 6 months after the start of chemotherapy) was compatible with disease progression, with dimensional increase on the previously known lung metastases. Second-line palliative treatment with pegylated liposomal doxorubicin 40 mg/m^2^ every 4 weeks was started. The patient completed only three cycles of therapy before stopping the treatment due to pulmonary disease progression. Third-line palliative treatment was started with gemcitabine 1,000 mg/m^2^ once weekly for 3 weeks of each 28-day cycle, which was stopped after six cycles, again due to progression on the lung metastases. Fourth-line treatment was started with docetaxel 50 mg/m^2^ weekly, with no response on the CT scan after five cycles. At this stage it was decided to start treatment with pazopanib, a tyrosine kinase inhibitor targeting VEGFR. Once again, the drug was stopped after only 3 months due to pulmonary disease progression. Taking into consideration the deterioration of the patient’s performance status, a shared decision was reached to stop chemotherapy and focus on optimal supportive care. The patient died due to respiratory failure, secondary to pulmonary disease progression, 29 months after the diagnosis.

## DISCUSSION

Thyroid angiosarcoma is a rare malignancy that occurs more frequently in females, mostly over the age of 60 (2). Most of the reported cases occur in patients living in the mountainous alpine region, where iodine deficiency and endemic goiter are common. This report describes a very rare case of a non-alpine TAS that occurred in a patient with no previous history of goiter. From the start, the thyroid nodule presented with an aggressive behavior, as well as rapid growth and compressive symptoms. The ultrasound findings raised the suspicion of malignancy, and the FNAC was inconclusive. The cytologic diagnosis in these cases can be quite challenging due to cellular paucity, necrosis and lack of awareness given the rarity of this disease (4). In this case, the biopsy of the thyroid lesion was highly informative and indicative of a vascular malignancy. We then performed a total thyroidectomy with lymph node dissection, both for histological confirmation of diagnosis, and for symptomatic relief, due to the rapid growth and compressive symptoms. Histology of the resection specimen confirmed the initial diagnosis of TAS. Both the morphologic aspects and the immunophenotype rendered – positivity for endothelial markers CD31, CD34 – confirmed the vascular/endothelial histogenesis. When in doubt, Freund’s leukemia integration site 1 (FLI1) and von Willebrand’s factor VIII-related antigen (factor VII-RA) can also be very useful for the diagnosis of TAS (2). Thyroglobulin staining was negative. In the past, TAS was a controversial entity because it was considered a vascular mutation of anaplastic carcinoma and not a true sarcoma (3). However, according to the last World Health Organization (WHO) classification of tumors of endocrine organs, TAS is considered a distinct entity included in the group of vascular tumors (8). Due to its rarity and the small number of reported cases, no gold standard of treatment has been established up to this moment. Radical surgery in a very early stage seems to be curative in some cases, and some authors also reported promising results with adjuvant radiotherapy (2,9). Local recurrence is a common occurrence in soft tissue sarcoma (10). In a series reported by Lahat and cols., the local recurrence was an independent predictor of adverse outcome in patients who were submitted to a complete resection of primary soft tissue sarcoma (11). However, the propensity or significance of local recurrence regarding angiosarcoma is less clear (10). In a series reported by Espat and cols., 16% of angiosarcoma patients developed local recurrence, less than the reported rates of recurrence in other soft tissues sarcomas (10,12). Locally recurrent angiosarcoma is often treatable, and complete resection remains the best means to achieve control and potentially prolonged survival (10). In this particular case, radiation therapy played an important role in stabilizing local recurrences. According to a systematic review by De Felice and cols., the choice of chemotherapy regimens usually favors the use of taxanes, epirubicin, doxorubicin or ifosfamide but without a clear benefit (2). In the reported case, we also used pazopanib (a tyrosine kinase inhibitor targeting the VEGFR often used in other soft tissue sarcomas) but with no response. Unfortunately, TAS still has a poor prognosis, with survival rates being limited to just a few months after diagnosis. In our case, we achieved a survival of 29 months after diagnosis, which contributes to the notion that the diagnosis and treatment of patients with rare disease should be discussed in multidisciplinary groups, encompassing the possibility of locoregional treatments with surgery/radiotherapy or systemic treatments with chemotherapy/targeted agents. The presence of capsular invasion and distant organs metastases at diagnosis are the strongest negative prognostic factors. More evidence is needed to optimize the management of this disease (2). Additional reports of this rare neoplasm can provide new data that might contribute to furthering our knowledge about TAS and hopefully improving the outcome of these patients.
